# Degenerate Cauchy numbers of the third kind

**DOI:** 10.1186/s13660-018-1626-x

**Published:** 2018-02-05

**Authors:** Sung-Soo Pyo, Taekyun Kim, Seog-Hoon Rim

**Affiliations:** 1 Department of mathematics Education, Silla University, Busan, Republic of Korea; 2 Department of Mathematics, Kwangwoon University, Seoul, Republic of Korea; 3 Department of Mathematics Education, Kyungpook National University, Daegu, Republic of Korea

**Keywords:** 05A19, 11B75, 11B73, Cauchy numbers, Degenerate Cauchy numbers, Degenerate Cauchy numbers of the third kind

## Abstract

Since Cauchy numbers were introduced, various types of Cauchy numbers have been presented. In this paper, we define degenerate Cauchy numbers of the third kind and give some identities for the degenerate Cauchy numbers of the third kind. In addition, we give some relations between four kinds of the degenerate Cauchy numbers, the Daehee numbers and the degenerate Bernoulli numbers.

## Introduction

It is well known that the *Cauchy numbers* (or *the Bernoulli numbers of the second kind*), denoted by $C_{n}$, are derived from the integral as follows:

1 $$\begin{aligned} \int_{0}^{1} (1+t)^{x} \,dx &= \frac{t}{\log(1+t)} \\ &= \sum_{n=0}^{\infty} C_{n} \frac{t^{n}}{n!}. \end{aligned}$$

The Cauchy numbers play a very important role in the study of mathematical physics (see [1] and [2]). Various characteristics of the Cauchy numbers can be found in [3–7]. For other definitions and properties of the Cauchy numbers, the reader can consult [8, pp. 293–294], [9] and [10, p. 114].

In [11], Kim introduced a new class of numbers which are called the *degenerate Cauchy numbers*, denoted by $C_{n, \lambda}$, as follows:

2 $$\begin{aligned} \int_{0}^{1} \bigl(1+ \log(1+ \lambda t)^{\frac{1}{\lambda}}\bigr)^{x} \,dx &= \frac{\frac{1}{\lambda} \log(1+ \lambda t)}{\log(1+\frac{1}{\lambda} \log(1+ \lambda t))} \\ &= \sum_{n=0}^{\infty} C_{n,\lambda} \frac{t^{n}}{n!}\quad (\lambda>0). \end{aligned}$$

From (2), we note that

3 $$ \lim_{\lambda\rightarrow0} \frac{\frac{1}{\lambda} \log(1+ \lambda t)}{\log(1+\frac{1}{\lambda} \log(1+ \lambda t))} = \frac{t}{\log(1+t)}. $$

The degenerate Cauchy numbers of the second kind, denoted by $C_{n, \lambda,2}$, are introduced in [12] as follows:

4 $$ \frac{t}{\log(1+\frac{1}{\lambda} \log(1+ \lambda t))} = \sum_{n=0}^{\infty} C_{n,\lambda,2} \frac{t^{n}}{n!}. $$

As with equation (3), we know that

5 $$ \lim_{\lambda\rightarrow0} \frac{t}{\log(1+\frac{1}{\lambda} \log(1+ \lambda t))} = \frac{t}{\log(1+t)}. $$

The degenerate Cauchy numbers have a lot of interesting properties. One of them is a relation between the Cauchy numbers and the degenerate Cauchy numbers:

$$ C_{n, \lambda}= \sum_{l=0}^{\infty} \lambda^{n-l} S_{1}(n, l) C_{l}, $$

where $S_{1}(n, k)$ is the Stirling numbers of the first kind.

In [12], Kim proved that the following identity holds:

$$ C_{n,\lambda} = \sum_{m=0}^{n} \binom{n}{m} \lambda^{n-m} D_{n-m} C_{m, \lambda, 2}, $$

where $D_{n}$ are the Daehee numbers which are defined by the generating function to be

6 $$ \frac{\log(1+t)}{t} = \sum_{n=0}^{\infty} D_{n} \frac{t^{n}}{n!}\quad \text{(see [13--17])}. $$

Let us take note of the following:

7 $$ \int_{0}^{1} \bigl( 1+ \lambda\log(1+t) \bigr)^{\frac{x}{\lambda}} \,dx = \frac{ \lambda ( (1+\lambda\log(1+t))^{\frac{1}{\lambda}} -1 )}{\log( 1+ \lambda\log(1+t))}\quad (\lambda>0). $$

In equation (7), we know that

8 $$ \lim_{\lambda\rightarrow0} \frac{ \lambda ( (1+\lambda \log(1+t))^{\frac{1}{\lambda}} -1 )}{\log( 1+ \lambda \log(1+t))} = \frac{t}{\log(1+t)}. $$

From (8), equation (7) must be related to the Cauchy numbers. We define *the degenerate Cauchy numbers of the third kind*, denoted by $C_{n,\lambda,3}$, by the generating function

9 $$ \frac{ \lambda ( (1+\lambda\log(1+t))^{\frac{1}{\lambda}} -1 )}{\log( 1+ \lambda\log(1+t))} = \sum_{n=0}^{\infty} C_{n, \lambda,3} \frac{t^{n}}{n!}. $$

As the definition of the degenerate Cauchy numbers of the second kind comes from the definition of those of the first kind, we define *the degenerate Cauchy numbers of the forth kind* by the generating function as follows:

10 $$ \frac{ \lambda t}{\log( 1+ \lambda\log(1+t))} = \sum_{n=0}^{\infty} C_{n, \lambda,4} \frac{t^{n}}{n!}. $$

As *λ* goes to zero in equation (10), the generating function of the degenerate Cauchy numbers of the forth kind goes to the generating function of the Cauchy numbers, that is,

11 $$ \lim_{\lambda\rightarrow0} \frac{ \lambda t}{\log( 1+ \lambda \log(1+t))} = \frac{t}{\log(1+t)}. $$

Very recently, a study on the degenerate Cauchy polynomials and numbers of the fourth kind was conducted by Pyo [18].

Equations (3), (5), (8) and (11) give us

12 $$\begin{aligned} \lim_{\lambda\rightarrow0} C_{n, \lambda} = \lim _{\lambda \rightarrow0} C_{n, \lambda,2}= \lim_{\lambda\rightarrow0} C_{n, \lambda,3}= \lim_{\lambda \rightarrow0} C_{n, \lambda,4} = C_{n}. \end{aligned}$$

When $n=0$, we know that

13 $$ C_{0} = C_{0, \lambda}= C_{0, \lambda,2} = C_{0, \lambda,3} = C_{0, \lambda,4} = 1. $$

**Figure 1:**
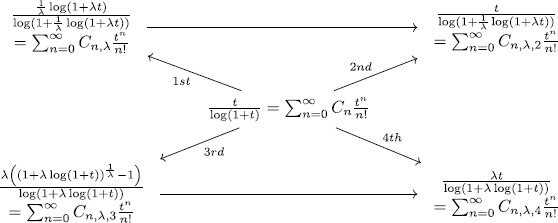
Four kinds of degenerate Cauchy numbers

Figure 1 shows the four kinds of degenerate Cauchy numbers.

Throughout this article, we develop research in the scope of real numbers. It is necessary to check the range of *λ*. From (2) and (9), depending on the range of the logarithm function, *λ* must be greater than 0. The limits (3) and (8) indicate that *λ* does not matter if it is zero.

When *λ* goes to infinity, the generating functions of both the degenerate Cauchy numbers and those of the third kind, (2) and (9), converge to 1, but those of the second kind and the fourth kind, (4) and (10), are divergent.

From the argument of the range of *λ*, we know that *λ* could be any non-zero positive real number. From now on, we consider *λ* to be a certain positive real number.

In this paper, we give some identities for the degenerate Cauchy numbers of the third kind, and give some relations between the degenerate Cauchy numbers of the third kind and the degenerate Cauchy numbers of other kinds.

## The degenerate Cauchy numbers of the third kind

From the definition of the degenerate Cauchy numbers of the third kind, (7) and (9), we have

14 $$\begin{aligned} \sum_{n=0}^{\infty} C_{n,\lambda,3} \frac{t^{n}}{n!} &= \int_{0}^{1} \bigl( 1+ \lambda\log(1+t) \bigr)^{\frac{x}{\lambda}} \,dx \\ &= \int_{0}^{1} e^{ {\frac{x}{\lambda}\log( 1+ \lambda\log(1+t))}} \,dx \\ &= \sum_{m=0}^{\infty} \lambda^{-m} \int_{0}^{1} x^{m} \,dx \frac{1}{m!} \bigl( \log \bigl( 1+ \lambda\log(1+t) \bigr) \bigr)^{m} \\ &= \sum_{m=0}^{\infty} \frac{\lambda^{-m}}{m+1} \sum _{l=m}^{\infty} S_{1} (l,m) \lambda^{l} \frac{( \log(1+t))^{l}}{l!} \\ &= \sum_{l=0}^{\infty} \sum _{m=0}^{l} \frac{\lambda^{l-m}}{m+1} S_{1} (l,m) \lambda^{l} \frac{( \log(1+t))^{l}}{l!} \\ &= \sum_{l=0}^{\infty} \Biggl( \sum _{m=0}^{l} \frac{\lambda^{l-m}}{m+1} S_{1} (l,m) \lambda^{l} \Biggr) \sum_{n=l}^{\infty} S_{1} (n,l) \frac {t^{n}}{n!} \\ &= \sum_{n=0}^{\infty} \sum _{l=0}^{n} \sum_{m=0}^{l} \frac{\lambda^{l-m}}{m+1} S_{1} (l,m) S_{1} (n,l) \frac{t^{n}}{n!}. \end{aligned}$$

From (14), we have the following theorem.

### Theorem 1

*For any integer* $n \ge0$ *and real* $\lambda>0$,

15 $$ C_{n,\lambda,3} = \sum_{0 \le l \le n} \sum _{0 \le m \le l} \frac{\lambda^{l-m}}{m+1} S_{1} (l,m) S_{1} (n,l). $$

When *λ* goes to zero in equation (15), the right-hand side of equation (15) remains only if $m=l$. Thus we have

$$ \lim_{\lambda\rightarrow0} C_{n,\lambda,3} = \sum _{l=0}^{n} \frac{S_{1} (n,l)}{l+1} = C_{n}. $$

The *n*th falling factorial of *x*, denoted by $(x)_{n}$, is given by

16 $$ (x)_{0} = 1,~ (x)_{n} = x (x-1) \cdots(x-n+1). $$

In [19], Carlitz introduced *λ*-analogue of falling factorials, and in [20], Kim presented several results regarding it. The *λ*-analogue of falling factorials is defined as follows:

17 $$ (x)_{0,\lambda} = 1,~ (x)_{n, \lambda} = x (x-\lambda) (x- 2 \lambda ) \cdots\bigl(x-(n-1)\lambda\bigr). $$

Note that $\lim_{\lambda\rightarrow1 } (x)_{n, \lambda} = (x)_{n}$, and $\lim_{\lambda\rightarrow0 } (x)_{n, \lambda} = x^{n}$.

The Stirling numbers of the first kind are defined as

18 $$ (x)_{n} = \sum_{l=0}^{n} S_{1} (n,l) x^{l}, $$

where $S_{1} (n, l), (n,l \ge0)$ are called the Stirling numbers of the first kind.

From (17) and (18), Kim defined the *λ*-analogue of the Stirling numbers of the first kind as follows:

19 $$ (x)_{n,\lambda} = \sum_{l=0}^{n} S_{1, \lambda} (n,l)x^{l},\quad (n \ge0). $$

The coefficients $S_{1,\lambda}(n,l)$ on the right-hand side of (19) are called the *λ*-analogue of the Stirling numbers of the first kind.

We note that

20 $$\begin{aligned} \biggl( \frac{1}{\lambda} \biggr)_{l} & = \frac{1}{\lambda} \biggl( \frac{1}{\lambda} -1 \biggr) \biggl( \frac{1}{\lambda} - 2 \biggr) \cdots \biggl( \frac{1}{\lambda}-l+1 \biggr) \\ &= \frac{1}{\lambda^{l+1}} ( 1- \lambda) ( 1-2 \lambda) \cdots\bigl( 1- (l-1) \lambda \bigr) \\ &= \lambda^{-l} (1)_{l,\lambda}, \end{aligned}$$

and

21 $$ (1+ t)^{\frac{1}{\lambda}} = 1+ \lambda^{-1}(1)_{1,\lambda} \frac{t}{1!} + \lambda^{-2}(1)_{2,\lambda} \frac{t^{2}}{2!} + \cdots. $$

By replacing *t* by $e^{\frac{t}{\lambda}} -1$ in the first line of equation (14), we get

22 $$\begin{aligned} \sum_{n=0}^{\infty} C_{n, \lambda, 3} \frac{1}{n!} \bigl( e^{\frac{t}{\lambda}} -1 \bigr)^{n} &= \int_{0}^{1} \bigl( 1+ \lambda \log \bigl(1+e^{\frac{t}{\lambda}} -1 \bigr) \bigr)^{\frac{x}{\lambda}} \,dx \\ &= \int_{0}^{1} ( 1+ t )^{\frac{x}{\lambda}}\,dx \\ &= \frac{\lambda}{\log(1+t)} \bigl( (1+ t)^{\frac{1}{\lambda}} -1 \bigr). \end{aligned}$$

From (20) and (21), we obtain

23 $$\begin{aligned} \frac{\lambda}{\log(1+t)} \bigl( (1+ t)^{\frac{1}{\lambda}} -1 \bigr) &= \frac{\lambda}{\log(1+t)} \sum_{l=1}^{\infty} \biggl( \frac{1}{\lambda} \biggr)_{l} \frac{t^{l}}{l!} \\ &= \lambda\frac{ t}{\log(1+t)} \sum_{l=0}^{\infty} \biggl( \frac{1}{\lambda} \biggr)_{l+1} \frac{t^{l}}{(l+1)!} \\ &= \sum_{m=0}^{\infty} C_{m} \frac{t^{m}}{m!} \sum_{l=0}^{\infty} \frac{(1)_{l+1,\lambda}}{\lambda^{l}(l+1)} \frac {t^{l}}{l!} \\ &= \sum_{n=0}^{\infty} \sum _{l=0}^{n} \binom{n}{l} C_{n-l} \frac{( 1)_{l+1,\lambda}}{\lambda^{l}(l+1)} \frac{t^{n}}{n!}. \end{aligned}$$

From (22) and (23), we have

24 $$ \int_{0}^{1} \bigl( 1+ \lambda\log \bigl(1+e^{\frac{t}{\lambda}} -1 \bigr) \bigr)^{\frac{x}{\lambda}} \,dx = \sum _{n=0}^{\infty} \sum_{l=0}^{n} \binom{n}{l} C_{n-l} \frac{( 1 )_{l+1,\lambda}}{\lambda^{l}(l+1)} \frac{t^{n}}{n!}. $$

The left-hand side in equation (22) becomes

25 $$\begin{aligned} \sum_{l=0}^{\infty} C_{l, \lambda, 3} \frac{1}{l!} \bigl( e^{\frac{t}{\lambda}} -1 \bigr)^{l} & = \sum_{l=0}^{\infty} C_{n, \lambda, 3} \frac{1}{l!} \sum_{n=l}^{\infty} S_{2} (n, l) \lambda^{-n} \frac{t^{n}}{n!} \\ & = \sum_{n=0}^{\infty} \sum _{l=0}^{n} C_{n, \lambda, 3} S_{2} (n, l) \lambda^{-n} \frac{t^{n}}{n!}, \end{aligned}$$

where $S_{2} (n,l)$ denotes the Stirling number of the second kind.

We note that

26 $$ \biggl( \frac{x}{ \lambda} \biggr)_{l} = \lambda^{-l} (x)_{l, \lambda}. $$

Applying (26), let us consider the left-hand side of equation (24) in different way with (22):

27 $$\begin{aligned} \int_{0}^{1} \bigl( 1+ \lambda\log \bigl(1+e^{\frac{t}{\lambda}} -1 \bigr) \bigr)^{\frac{x}{\lambda}} \,dx & = \int_{0}^{1} ( 1+t )^{\frac{x}{\lambda}} \,dx \\ &= \int_{0}^{1} \sum_{n=0}^{\infty} \biggl( \frac{x}{ \lambda} \biggr)_{n} \frac{t^{n}}{n!} \,dx \\ &= \int_{0}^{1} \sum_{n=0}^{\infty} \lambda^{-n} (x)_{n, \lambda} \frac{t^{n}}{n!} \,dx \\ &= \sum_{n=0}^{\infty} \lambda^{-n} \sum_{k=n}^{\infty} S_{1,\lambda} (k,n) \int_{0}^{1} x^{k} \,dx \frac{t^{n}}{n!} \\ &= \sum_{n=0}^{\infty} \lambda^{-n} \sum_{k=n}^{\infty} S_{1,\lambda} (k,n) \int_{0}^{1}x^{k} \,dx \frac{t^{n}}{n!} \\ &= \sum_{n=0}^{\infty} \lambda^{-n} \sum_{k=n}^{\infty} \frac{S_{1,\lambda} (k,n)}{k+1} \frac{t^{n}}{n!}. \end{aligned}$$

From (24), (25) and (27), we have the following theorem.

### Theorem 2

*For any integer* $n \ge0$ *and real* $\lambda>0$,

28 $$ \sum_{0 \le l \le n} C_{n, \lambda, 3} S_{2} (n, l) = \sum_{0 \le l \le n} \binom{n}{l} C_{n-l}\frac{\lambda ^{n-l} ( 1)_{l+1,\lambda}}{l+1} = \sum_{n \le k \le\infty} \frac{S_{1,\lambda} (k,n)}{k+1}. $$

If *λ* goes to 0 in both sides of the first equality in equation (28), then the second term of equation (28) remains only if $l=n$. And $( 1)_{l+1,\lambda}$ goes to 1 if *λ* goes to 0. From (12), $C_{n, \lambda, 3}$ goes to $C_{n} $ if *λ* goes to 0. Therefore we get the following identity.

### Corollary 3

*For any integer* $n \ge0$,

$$ \frac{1}{n+1} = \sum_{0 \le l \le n} C_{n} S_{2} (n, l). $$

We note that $\lim_{\lambda\rightarrow0} S_{1,\lambda}(n,k) = \delta_{n,k}$, where $\delta_{n,k}$ denotes the Kronecker symbol [20]. Both sides of the second equation in (28) go to $\frac{1}{n+1}$ as *λ* goes to 0.

When $\lambda= 1$ in the first line of equation (22), the right-hand side becomes

29 $$\begin{aligned} \int_{0}^{1} ( 1+ t)^{x} \,dx &= \frac{ t}{\log( 1+ t) } \\ & = \sum_{n=0}^{\infty} C_{n} \frac{t^{n}}{n!}, \end{aligned}$$

and the left-hand side of equation (22) becomes

30 $$\begin{aligned} \sum_{l=0}^{\infty} C_{l, 1, 3} \frac{1}{l!} \bigl( e^{t} -1 \bigr)^{l} & = \sum_{l=0}^{\infty}C_{l, 1, 3} \sum_{n=l}^{\infty} S_{2} (n,l) \frac{t^{n}}{n!} \\ & = \sum_{n=0}^{\infty} \sum _{l=0}^{n}C_{l, 1, 3} S_{2} (n,l) \frac{t^{n}}{n!}. \end{aligned}$$

From (29) and (30), we have the following theorem.

### Theorem 4

*For any integer* $n \ge0$ *and real* $\lambda>0$,

$$ C_{n} = \sum_{0 \le l \le n} C_{l, 1, 3} S_{2} (n,l). $$

## Comparison between four kinds of the degenerate Cauchy numbers, the Daehee numbers and the degenerate Bernoulli numbers

It is well known that the degenerate Bernoulli numbers are defined by the generating function

31 $$ \frac{t}{(1+ \lambda t)^{\frac{1}{\lambda}} -1} = \sum_{n=0}^{\infty} \beta_{n,\lambda} \frac{t^{n}}{n!}. $$

We note that equation (31) is defined for all real-valued *λ*. So, in equation (31), there is no problem to switch *λ* into $\frac{1}{\lambda}$ as follows:

32 $$ \frac{t}{(1+ \frac{1}{\lambda} t)^{{\lambda}} -1} = \sum_{n=0}^{\infty} \beta_{n,\frac{1}{\lambda}} \frac{t^{n}}{n!}. $$

In equation (31), the left-hand side equation is divergent as *λ* goes to infinity. So, the left-hand side in equation (32) is divergent as *λ* goes to 0. We need to point out that if *λ* does not equal 0, equation (32) is meaningful.

By replacing *t* with $\log(1+t)$, equation (31) becomes

33 $$\begin{aligned} \frac{\log(1+t)}{(1+ \lambda\log(1+t))^{\frac{1}{\lambda}} -1} &= \sum_{k=0}^{\infty} \beta_{k,\lambda} \frac{\log(1+t)^{k}}{k!} \\ &= \sum_{k=0}^{\infty} \beta_{k,\lambda} \sum _{n=k}^{\infty} S_{1}(n,k) \frac{t^{n}}{n!} \\ &= \sum_{n=0}^{\infty} \sum _{k=0}^{n} \beta_{k,\lambda} S_{1}(n,k) \frac{t^{n}}{n!}. \end{aligned}$$

Using similar process to (33) in equation (32), we get

34 $$ \frac{\log(1+t)}{(1+ \frac{1}{\lambda} \log(1+t))^{{\lambda}} -1} = \sum_{n=0}^{\infty} \sum_{k=0}^{n} \beta_{k,\frac{1}{\lambda}} S_{1}(n,k) \frac{t^{n}}{n!}. $$

We derive the following (35) by using (33):

35 $$\begin{aligned} &\frac{ \lambda\log(1+t)}{\log(1+ \lambda\log(1+t))} \\ & \quad = \frac{ \lambda ( (1 + \lambda\log(1+t))^{\frac{1}{\lambda}} -1 )}{\log(1+ \lambda\log(1+t))} \cdot \frac{\log(1+t)}{(1+ \lambda\log(1+t))^{\frac{1}{\lambda}}-1} \\ &\quad =\sum_{l=0}^{\infty} C_{l, \lambda,3} \frac{t^{l}}{l!}\sum_{m=0}^{\infty} \sum _{k=0}^{m} \beta_{k,\lambda} S_{1}(m,k) \frac{t^{m}}{m!} \\ &\quad =\sum_{n=0}^{\infty}\sum _{l=0}^{n} \sum_{k=0}^{l} \binom{n}{l} C_{n-l, \lambda,3} \beta_{k,\lambda} S_{1}(l,k) \frac{t^{n}}{n!}. \end{aligned}$$

In equation (2), the definition of the degenerate Cauchy numbers of the first kind, by converting *λ* to $\frac{1}{\lambda}$, we have

36 $$ \frac{ {\lambda} \log ( 1+ \frac{t}{\lambda} )}{\log(1+ { \lambda} \log(1+\frac{t}{\lambda}))} = \sum_{n=0}^{\infty} C_{n,\lambda^{-1}} \frac{t^{n}}{n!}\quad (\lambda>0). $$

We know that equation (36) goes to the generating function of the Cauchy numbers as *λ* goes to infinity. Although *λ* is a constant real, it is necessary to check the new inspection by substituting the reciprocal of *λ*. It is not difficult to show that

37 $$ \lim_{\lambda\rightarrow0} \frac{ {\lambda} \log ( 1+ \frac{t}{\lambda} )}{\log(1+ { \lambda} \log(1+\frac{t}{\lambda}))} = 1. $$

Equation (37) shows that $C_{n,\frac{1}{\lambda}}$ converges to 1 as *λ* goes to 0 only if $n=0$, and converge to 0 when $n \ge1$. Equation (36) is meaningful for nonnegative real *λ*. The following equation (38) can be obtained by substituting *λt* instead of *t* in equation (36):

38 $$ \frac{ {\lambda} \log ( 1+ t )}{\log(1+ { \lambda} \log(1+t))} = \sum_{n=0}^{\infty} C_{n,\lambda^{-1}} \lambda^{n} \frac{t^{n}}{n!}. $$

Using (35) and (38), we get the following theorem.

### Theorem 5

*For any integer* $n \ge0$ *and real* $\lambda>0$,

$$ C_{n,\frac{1}{\lambda}} = \sum_{0 \le l \le n} \sum _{0 \le k \le l} \binom{n}{l} \frac{C_{n-l, \lambda,3} \beta _{k,\lambda} S_{1}(l,k)}{\lambda^{n}}. $$

Let $G=G(t) = \frac{(1+ \lambda t)^{\frac{1}{\lambda}}-1}{t}$, then

39 $$\begin{aligned} G & = \frac{(1+ \lambda t)^{\frac{1}{\lambda}}-1}{t} \\ & = \frac{1}{t} \sum_{k=1}^{\infty} \biggl( \frac{1}{\lambda} \biggr)_{k} \lambda^{k} \frac{t^{k}}{k!} \\ &= \sum_{k=1}^{\infty} (1)_{k,\lambda} \frac{t^{k-1}}{k!} \\ &= \sum_{k=0}^{\infty} \frac{ (1)_{k+1, \lambda}}{k+1} \frac{t^{k}}{k!}. \end{aligned}$$

By replacing *t* with $\log(1+t)$ in (39),

40 $$\begin{aligned} \frac{(1+ \lambda\log(1+t))^{\frac{1}{\lambda}}-1}{\log(1+t)} &= \sum_{k=0}^{\infty} \frac{ (1)_{k+1,\lambda}}{k+1} \frac{(\log(1+t))^{k}}{k!} \\ &= \sum_{k=0}^{\infty} \frac{ (1)_{k+1,\lambda}}{k+1} \sum _{n=k}^{\infty} S_{1} (n,k) \frac{t^{n}}{n!} \\ &= \sum_{n=0}^{\infty}\sum _{k=0}^{n} \frac{ (1)_{k+1,\lambda}}{k+1} S_{1} (n,k) \frac{t^{n}}{n!}. \end{aligned}$$

We note that

41 $$\begin{aligned} &\frac{ \lambda ( \log(1 + \lambda \log(1+t))^{\frac{1}{\lambda}} -1 )}{\log(1+ \lambda\log(1+t))} \\ &\quad = \frac{ \lambda \log(1+t)}{\log(1+ \lambda\log(1+t))} \cdot \frac{(1+ \lambda\log(1+t))^{\frac{1}{\lambda}}-1}{\log(1+t)} \\ &\quad=\sum_{l=0}^{\infty} C_{l, \frac{1}{\lambda}} \lambda^{l} \frac{t^{l}}{l!} \sum_{n=0}^{\infty} \sum_{k=0}^{n} \frac{ (1)_{k+1,\lambda}}{k+1} S_{1} (n,k) \frac{t^{n}}{n!} \\ &\quad =\sum_{n=0}^{\infty} \sum _{l=0}^{n}\sum_{k=0}^{l} \binom{n}{l} \frac{ C_{n-l, \frac{1}{\lambda}} \lambda^{n-l} (1)_{k+1,\lambda}}{k+1} S_{1} (l,k) \frac{t^{n}}{n!}. \end{aligned}$$

From (40) and (41), we have the following theorem.

### Theorem 6

*For any integer* $n \ge0$ *and real* $\lambda>0$,

$$ C_{n,\lambda,3} = \sum_{0 \le l \le n}\sum _{ 0 \le k \le l} \binom{n}{l} \frac{ C_{n-l, \frac{1}{\lambda}} \lambda^{n-l} (1)_{k+1,\lambda}}{k+1} S_{1} (l,k). $$

Consider the following equation (42) which is obtained from the definition of the degenerate Cauchy numbers of the third kind, equation (9), by replacing *λ* with $\frac{1}{\lambda}$.

42 $$ \frac{\frac{1}{\lambda} ( ( 1+ \frac{1}{\lambda} \log(1+t))^{\lambda} ) -1 }{\log(1+\frac{1}{\lambda} \log(1+ t))} = \sum_{n=0}^{\infty} C_{n,\frac{1}{\lambda},3} \frac{t^{n}}{n!}. $$

As shown in equation (36), it is not difficult to know that

43 $$ \lim_{\lambda\rightarrow0} \frac{\frac{1}{\lambda} ( ( 1+ \frac{1}{\lambda} \log(1+t))^{\lambda} ) -1 }{\log(1+\frac{1}{\lambda} \log(1+ t))} = 1. $$

Just like $C_{n,\frac{1}{\lambda}}$, we can see that $C_{n,\frac{1}{\lambda},3}$ converges to 1 as *λ* goes to 0 only if $n=0$, and it converges to 0 when $n \ge1$ from equation (43).

We note that

44 $$\begin{aligned} &\frac{t}{\log(1+ \frac{1}{\lambda} \log(1+ {\lambda}{t}))} \\ & \quad= \frac{\frac{1}{\lambda} ( ( 1+ \frac{1}{\lambda} \log(1+ {\lambda} t))^{\lambda} ) -1 }{\log(1+ \frac{1}{\lambda} \log(1+ {\lambda}{t}))} \frac{\log(1+{\lambda}{t})}{ ( ( 1+ \frac{1}{\lambda} \log(1+{\lambda}t))^{\lambda} ) -1 } \\ & \qquad{}\times\frac{{\lambda}{t}}{ \log(1+{\lambda}{t})}. \end{aligned}$$

Applying (42), (34) and (1) respectively in equation (44), we have the following:

45 $$\begin{aligned} & \sum_{n=0}^{\infty} C_{n,{\lambda},2} \frac{t^{n}}{n!} \\ &\quad= \Biggl( \sum_{k=0}^{\infty} C_{k,\frac{1}{\lambda},3} \lambda^{k} \frac{t^{k}}{ n!} \Biggr) \Biggl( \sum _{l=0}^{\infty} \sum_{p=0}^{l} \beta_{p,\frac{1}{\lambda}} S_{1}(l,p) \lambda^{l} \frac{t^{l}}{ l!} \Biggr) \Biggl( \sum_{m=0}^{\infty} C_{m} \lambda^{m} \frac{t^{m}}{ m!} \Biggr) \\ &\quad= \Biggl( \sum_{k=0}^{\infty} C_{k,\frac{1}{\lambda},3} \lambda^{k} \frac{t^{k}}{ n!} \Biggr) \Biggl(\sum _{m=0}^{\infty} \sum_{l=0}^{m} \sum_{p=0}^{m-l}\binom{m}{l} \beta_{p,\frac{1}{\lambda}} S_{1}(l,p) {C_{m-l}} { \lambda^{m}} \frac{t^{m}}{ m!} \Biggr) \\ &\quad= \sum_{n=0}^{\infty} \Biggl( \sum _{m=0}^{n} \sum_{l=0}^{m} \sum_{p=0}^{m-l}\binom{n}{m} \binom{m}{l}{\lambda^{n}} C_{n-m,\frac{1}{\lambda},3} {\beta_{p,\frac{1}{\lambda}} S_{1}(m-l,p) C_{m-l}} \Biggr) \frac{t^{n}}{ n!}. \end{aligned}$$

From (45), we get the following identity.

### Theorem 7

*For any integer* $n \ge0$ *and real* $\lambda>0$,

$$ C_{n,{\lambda},2} = \sum_{0 \le m \le n} \sum _{0 \le l \le m} \sum_{0 \le p \le m-1} \binom{n}{m}\binom{m}{l}{\lambda^{n}} C_{n-m,\frac{1}{\lambda},3} { \beta_{p,\frac{1}{\lambda}} S_{1}(m-l,p) C_{m-l}}. $$

The generating function of the degenerate Cauchy numbers of the third kind substituting $\frac{t}{\lambda} $ instead of values *t* can be developed as follows:

46 $$\begin{aligned} & \frac{\lambda ( (1+\lambda \log(1+\frac{t}{\lambda}))^{\frac{1}{\lambda}} -1 ) }{\log(1+{\lambda} \log(1+ \frac{t}{\lambda}))} \\ &\quad =\frac{t}{\log(1+{\lambda} \log(1+ \frac{t}{\lambda}))} \frac{ ( (1+\lambda \log(1+\frac{t}{\lambda}))^{\frac{1}{\lambda}} -1 )}{ \log (1+\frac{t}{\lambda})} \\ &\qquad {}\times\frac{ \log(1+\frac{t}{\lambda})}{\frac{t}{\lambda}}. \end{aligned}$$

By a similar process to (45), applying (4), (40) and (6) respectively in this case, we have

47 $$\begin{aligned} &\sum_{n=0}^{\infty} C_{n,\lambda,3} \frac{t^{n}}{\lambda^{n} n!} \\ &\quad= \Biggl( \sum_{k=0}^{\infty} C_{k,\frac{1}{\lambda},2} \frac{t^{k}}{k!} \Biggr) \Biggl( \sum_{l=0}^{\infty} \sum_{p=0}^{l} \frac{ (1)_{p+1,\lambda}}{p+1} \frac{S_{1} (l,p)}{ \lambda^{l}} \frac{t^{l}}{l!} \Biggr) \Biggl( \sum _{m=0}^{\infty} \frac{D_{m}}{\lambda^{m}} \frac{t^{m}}{m!} \Biggr) \\ &\quad= \Biggl( \sum_{k=0}^{\infty} C_{k,\frac{1}{\lambda},2} \frac{t^{k}}{k!} \Biggr) \Biggl( \sum_{m=0}^{\infty} \sum_{l=m}^{\infty}\sum _{p=0}^{m-l} \frac{ (1)_{p+1,\lambda}}{p+1} \frac{D_{l} S_{1} (m-l,p)}{\lambda^{m}} \frac{t^{m}}{m!} \Biggr) \\ &\quad= \sum_{n=0}^{\infty} \Biggl( \sum _{k=0}^{n} \sum_{l=n-k}^{\infty} \sum_{p=0}^{n-k-l} C_{k,\frac{1}{\lambda},2} \frac{ (1)_{p+1,\lambda}}{p+1} \frac{D_{l} S_{1} (n-k-l,p)}{\lambda^{n-k}} \Biggr) \frac{t^{n}}{n!}. \end{aligned}$$

The coefficients of both sides in equation (47) give the following identity.

### Theorem 8

*For any integer* $n \ge0$ *and real* $\lambda>0$,

$$ C_{n, \lambda, 3} = \sum_{0 \le k \le n} \sum _{n-k \le l \le \infty}\sum_{0 \le p \le n-k-1} C_{k,\frac{1}{\lambda},2} \frac{ (1)_{p+1,\lambda}}{p+1} D_{l} S_{1} (n-k-l,p) \lambda^{k}. $$

## Results and discussion

In this paper, we define the degenerate Cauchy numbers of the third kind $C_{n,\lambda,3}$ which are obtained by the generating function $\frac{ \lambda ( (1+\lambda\log(1+t))^{\frac{1}{\lambda}} -1 )}{\log( 1+ \lambda\log(1+t))}$. The degenerate Cauchy numbers of the third kind $C_{n,\lambda,3}$ are explicitly determined by the Stirling numbers of the first kind (Theorem 1). We obtain the three identities about the Stirling numbers of the first kind and the Cauchy numbers by using $C_{n,\lambda,3}$, Theorem 2, Corollary 3 and Theorem 4. In addition, four relations between the degenerate Cauchy numbers of the third kind and other kinds of the degenerate Cauchy numbers (Theorems 5 and 6) as well as the degenerate Cauchy numbers of the second kind (Theorems 7 and 8) are presented.

## Conclusion

For real $\lambda>0$, the degenerate Cauchy numbers of the third kind $C_{n,\lambda,3}$ are obtained by the generating function $\frac{ \lambda ( (1+\lambda\log(1+t))^{\frac{1}{\lambda}} -1 )}{\log( 1+ \lambda\log(1+t))}$. If $\lambda>0$ goes to 0, then the generating function of the degenerate Cauchy numbers of the third kind converges to the generating function of the Cauchy numbers $\frac{t}{\log(1+t)}$. The Cauchy numbers can be said to be defined from the generating function of the degenerate Cauchy numbers of the third kind when $\lambda=0$. In this paper, we have shown that there are many interesting characteristics in the combinatorial number theory realm, even though $\lambda > 0$. Just as the Cauchy numbers play a very important role in the study of mathematical physics, we would like to see some applications to the study of mathematical physics of the degenerate Cauchy numbers of the third kind in the near future.

## References

[1] Kim D., Kim T. (2015). A note on poly-Bernoulli and higher-order poly-Bernoulli polynomials. Russ. J. Math. Phys..

[2] Kim T., Mansour T. (2014). Umbral calculus associated with Frobenius-type Eulerian polynomials. Russ. J. Math. Phys..

[3] Dolgy D.V., Kim D.S., Kim T., Mansour T. (2015). Degenerate poly-Cauchy polynomials. Appl. Math. Comput..

[4] Jeong J., Rim S.H., Kim B.M. (2015). On finite-times degenerate Cauchy numbers and polynomials. Adv. Differ. Equ..

[5] Kim D.S., Kim T., Dolgy D.V. (2015). Degenerate poly-Cauchy polynomials with a *q* parameter. J. Inequal. Appl..

[6] Simsek Y. (2017). Identities on the Changhee numbers and Apostol-type Daehee polynomials. Adv. Stud. Contemp. Math. (Kyungshang).

[7] Todorov P.C. (1993). On the Cauchy numbers. Facta Univ., Ser. Math. Inform..

[8] Comtet L. (1974). Advanced Combinatorics.

[9] Merlini D., Sprugnoli R., Verri M.C. (2006). The Cauchy numbers. Discrete Math..

[10] Roman S. (1984). The Umbral Calculus.

[11] Kim T. (2015). On degenerate Cauchy numbers and polynomials. Proc. Jangjeon Math. Soc..

[12] Kim T. (2017). Degenerate Cauchy numbers and polynomials of the second kind. Adv. Stud. Contemp. Math..

[13] Kim D.S., Kim T. (2017). Degenerate Laplace transform and degenerate gamma function. Russ. J. Math. Phys..

[14] Kim T. (2002). An invariant *p*-adic integral associated with Daehee numbers. Integral Transforms Spec. Funct..

[15] El-Desouky B.S., Mustafa A. (2016). New results on higher-order Daehee and Bernoulli numbers and polynomials. Adv. Differ. Equ..

[16] Jang G.W., Kim D.S., Kim T. (2017). Degenerate Changhee numbers and polynomials of the second kind. Adv. Stud. Contemp. Math..

[17] Jang G.W., Kwon J., Lee J.G. (2017). Some identities of degenerate Daehee numbers arising from nonlinear differential equation. Adv. Differ. Equ..

[18] Pyo, S.-S.: Degenerate Cauchy numbers and polynomials of the fourth kind. Adv. Stud. Contemp. Math. **28**(1) (2018, in press)

[19] Carlitz L. (1979). Degenerate Stirling, Bernoulli and Eulerian numbers. Util. Math..

[20] Kim T. (2017). *λ*-analogue of Stirling numbers of the first kind. Adv. Stud. Contemp. Math..

